# Host response transcriptomic analysis of Crimean-Congo hemorrhagic fever pathogenesis in the cynomolgus macaque model

**DOI:** 10.1038/s41598-021-99130-1

**Published:** 2021-10-06

**Authors:** Catherine E. Arnold, Charles J. Shoemaker, Darci R. Smith, Christina E. Douglas, Candace D. Blancett, Amanda S. Graham, Timothy D. Minogue

**Affiliations:** 1 Diagnostic Systems Division, U.S. Army Medical Research Institute of Infectious Diseases, Fort Detrick, MD USA; 2 Virology Division, U.S. Army Medical Research Institute of Infectious Diseases, Fort Detrick, MD USA; 3 Present Address: Defense Threat Reduction Agency, Fort Belvoir, VA USA; 4 Present Address: Genomics and Bioinformatics Department, Biological Defense Research Directorate, Naval Medical Research Center, Fort Detrick, MD USA; 5 Present Address: Immunodiagnostics Department, Biological Defense Research Directorate, Naval Medical Research Center, Fort Detrick, MD USA; 6 Present Address: National Biodefense Analysis and Countermeasures Center, Fort Detrick, MD USA

**Keywords:** Viral host response, Viral infection, Infectious-disease diagnostics

## Abstract

Crimean-Congo hemorrhagic fever virus (CCHFV) is a highly pathogenic tick-borne RNA virus prevalent in Asia, Europe, and Africa, and can cause a hemorrhagic disease (CCHF) in humans with mortality rates as high as 60%. A general lack of both effective medical countermeasures and a comprehensive understanding of disease pathogenesis is partly driven by an historical lack of viable CCHF animal models. Recently, a cynomolgous macaque model of CCHF disease was developed. Here, we document the targeted transcriptomic response of non-human primates (NHP) to two different CCHFV strains; Afghan09-2990 and Kosova Hoti that both yielded a mild CCHF disease state. We utilized a targeted gene panel to elucidate the transcriptomic changes occurring in NHP whole blood during CCHFV infection; a first for any primate species. We show numerous upregulated genes starting at 1 day post-challenge through 14 days post-challenge. Early gene changes fell predominantly in the interferon stimulated gene family with later gene changes coinciding with an adaptive immune response to the virus. There are subtle differences between viral strains, namely duration of the differentially expressed gene response and biological pathways enriched. After recovery, NHPs showed no lasting transcriptomic changes at the end of sample collection.

## Introduction

Crimean-Congo hemorrhagic fever virus (CCHFV) is a tick-borne virus with a wide geographical distribution across Africa and Eurasia^1^. CCHFV, a member of the *Orthonairovirus* genus in the *Nairoviridae* family, has a tripartite, negative strand RNA genome comprised of small (S), medium (M), and large (L) segments. CCHFV is the most genetically diverse arthropod-borne virus and is currently divided into six clades based on M segment divergence^2^. Human infections occur primarily by tick (*Hyalomma)* bites or exposure to blood or bodily fluids from infected animals or CCHF patients^3,4^. After infection and an incubation period of 1–2 weeks, a disease state develops that can range in severity from a mild, non-specific febrile illness to severe, potentially fatal disease characterized by hemorrhaging and shock. Case fatality rates vary widely by geography and can range from as low as 5% to in excess of 60%^5,6^; likely reflecting disparities in healthcare systems, disease detection, and potentially differences in host and viral genetics. In 2018, the World Health Organization designated CCHFV as one of ten high priority emerging infectious diseases due to its epidemic emergence potential and lack of approved medical countermeasures^7^.

The absence of effective countermeasures is the result of multiple factors including the lack of an immunocompetent animal model that recapitulates human disease, the paucity of information regarding CCHF pathogenesis, and the requirement for a maximum biocontainment laboratory (BSL-4) to perform CCHF research. Currently, we have a fragmented timeline of events that lead to hemorrhagic fever in humans after CCHFV transmission. Patient data and animal studies suggest that after an initial local replication, the virus spreads systemically targeting the liver and endothelium causing a massive, dysregulated immune response culminating in hemorrhagic fever in some cases^8,9^. Overall, more insight is urgently needed regarding CCHFV pathogenesis in both humans and animal models.

Historically, immunocompetent animal models that recapitulate severe human CCHF disease remained elusive. Mouse models of CCHFV pathogenesis relying on either use transgenic or transient suppression of interferon signaling have only recently become available^10,11^. A CCHF convalescent mouse model using IFNAR^−/−^ mice showed elevated levels of the cytokines IL-6, TNF-α, IL-1β and IL-17^12^. Other studies showed CCL-2, IFN-γ, IL-1β, IL-6, IL-10, and TNF to be upregulated in a STAT1-deficient mouse model of infection^13^. Similarly, human infection show strong cytokine production, including IL-6, IL-8, IL-12, TNF-α, CSF2, IFN-γ, IL-10, CCL2, CCL3, CCL4, and CCL7^14–16^. It is worth noting, however, that most of the extant human data is limited to cytokine protein detection and not changes in the host transcriptome.

CCHFV infection in NHPs was previously thought to be refractory; likely a result of most historical testing being done with the reference strain IbAr 10,200, which yielded largely asymptomatic results across a range of doses, administration routes, and primate species^17,18^. More recently, Haddock et al. described a cynomolgus macaque model where an intravenous (IV) or combined IV and subcutaneous (SC) exposure with a European CCHFV isolate, Kosova Hoti (hereafter called Hoti) can cause disease with instances of morbidity severe enough to justify euthanasia^17^. These results appeared to recapitulate the disease progression seen in severe human CCHF cases^19,20^. Subsequently, we expanded upon this model by comparing Hoti strain pathogenesis in NHPs with that of a member of the central Asiatic clade, Afghan09-2990 (hereafter called Afg09), which was isolated from a fatal case involving a United States soldier in Afghanistan^21^. In that study, we reported a largely uniform and mild disease state across all challenged NHPs with limited differences seen between isolates^22^. In the present manuscript, we interrogated longitudinally collected blood samples from this mild disease phenotype NHP study and employed a targeted transcriptomic approach to examine host response pathways shared across clades of CCHFV virus. This will lead to a better understanding of CCHF disease pathogenesis in NHP models, and establish potential biomarker correlates that can be used in future investigations of CCHF in NHPs and humans.

## Results

### Clinical presentation

**Figure 1:**
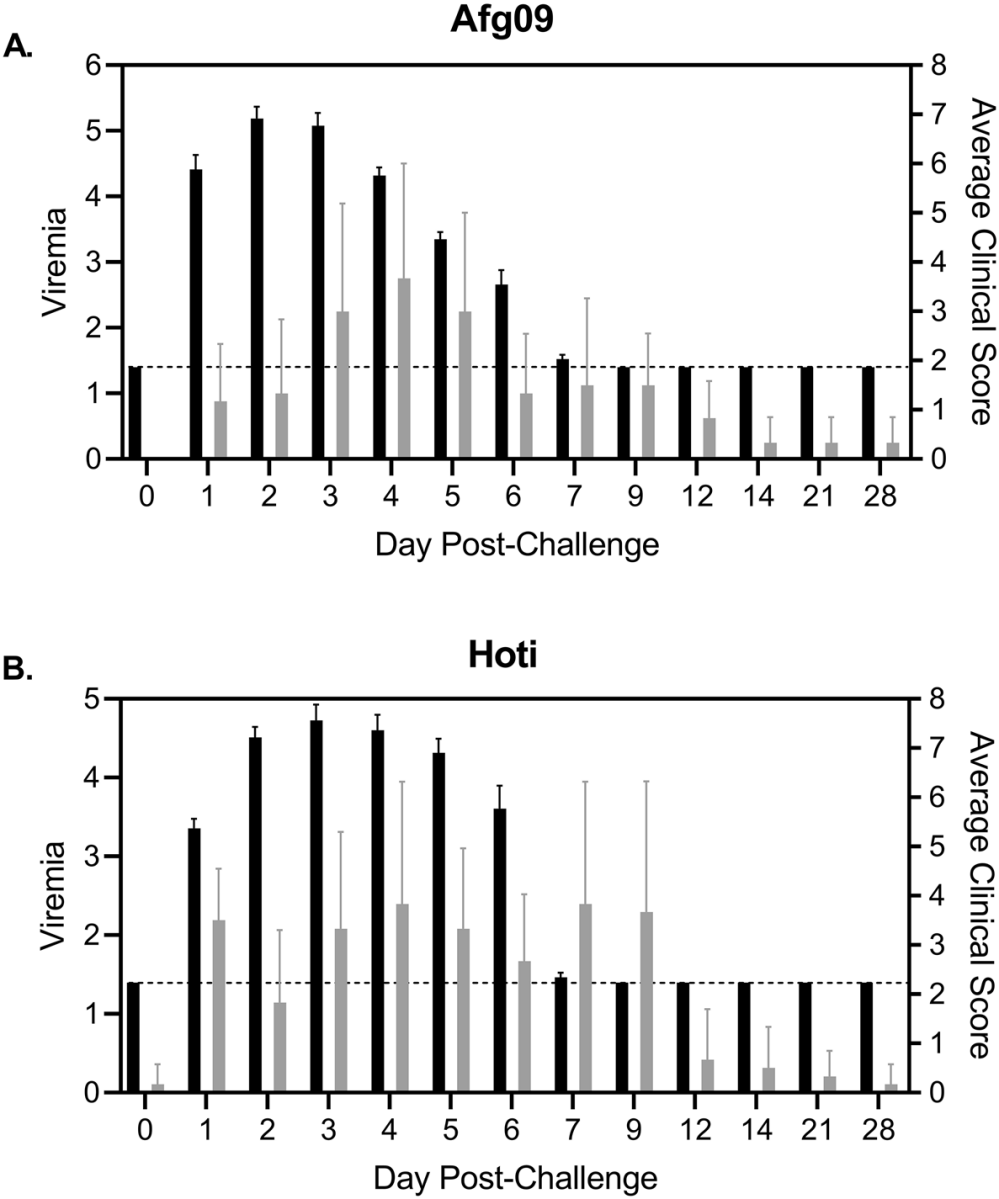
Viremia in NHPs for Afg09 and Hoti. Averaged challenge group viremia (black bars) and clinical scores (grey bars) for Afg09 (**A**) and Hoti (**B**). Whole blood viremia is expressed in PFU/mL (log10). Dashed lines indicate limit of detection for viremia assay. Error bars represent standard deviation. Aggregate NHP clinical data shown is from original study reported in^22^.

Previously detailed clinical disease profiles of cynomolgus macaques challenged with either Hoti or Afg09 isolates are summarized in Table 1^22^. Briefly, NHPs showed symptoms of CCHF disease that lasted approximately 7 days post challenge (DPC) prior to recovering and entering convalescence. The study concluded on day 28, and NHPs showed no signs of residual illness. Over the course of infection, NHPs in both challenge groups displayed fever, reduced platelets and lymphocytes, elevated liver enzymes ALT/AST, elevated serum cytokines, and in some cases rashes and orchitis. There were no statistical differences between viral strains in clinical presentation. Average viremia peaked 2 DPC for the Afg09 group and 3 DPC for the Hoti group, lasting until 7 DPC for both strains (Fig. 1A,B). Similarly, average group clinical scores largely resolved by 1 week post-challenge and were statistically non-significant from one another (Fig. 1A,B). After study completion, histopathological analysis revealed that a subset of the NHPs (4/6 in Afg09 group, 2/6 in Hoti group) had latent tuberculosis (TB) in the form of lung and liver granulomas; however, TB-positive NHPs showed no significant differences in any clinical parameter relative to TB negative NHPs.

**Table 1:** Summary of clinical findings from CCHFV Afg09 versus Hoti strain challenge study in cynomolgus macaques.

NHP	Viral isolate	Viremia	Clinical disease	Fever	Elevated serum cytokines	Thrombo-cytopenia	Lympho-penia	Elevated ALT/AST	Other symptoms	Anti-CCHFV antibody response
1	Hoti	Y	Y	+	Y	Y	Y	Y	Rash	Y
2	Hoti	Y	Y	++	Y	Y	Y	Y		Y
3	Hoti	Y	Y	++	Y	Y	Y	Y		Y
4	Hoti	Y	Y	+++	Y	Y	Y	Y		Y
5	Hoti	Y	Y	+++	Y	Y	Y	Y	Orchitis, rash	Y
6	Hoti	Y	Y	++	Y	Y	Y	Y	Orchitis, rash	Y
7	Afg09	Y	Y	++	Y	Y	Y	Y		Y
8	Afg09	Y	Y	++	Y	Y	Y	Y		Y
9	Afg09	Y	Y	++	Y	Y	Y	Y	Rash	Y
10	Afg09	Y	Y	++	Y	Y	Y	Y		Y
11	Afg09	Y	Y	++	Y	Y	Y	Y		Y
12	Afg09	Y	Y	+	Y	Y	Y	Y	Orchitis	Y

For fever data, 100 h or less of fever above baseline (+), 100–200 h above baseline (++), and 200 h or more above baseline (+++). IgG antibody response measured against both CCHFV Gn and nucleocapsid proteins. Clinical data summarized and reported as previously described in Smith et al.^22^.

### Differential gene expression trends

**Figure 2:**
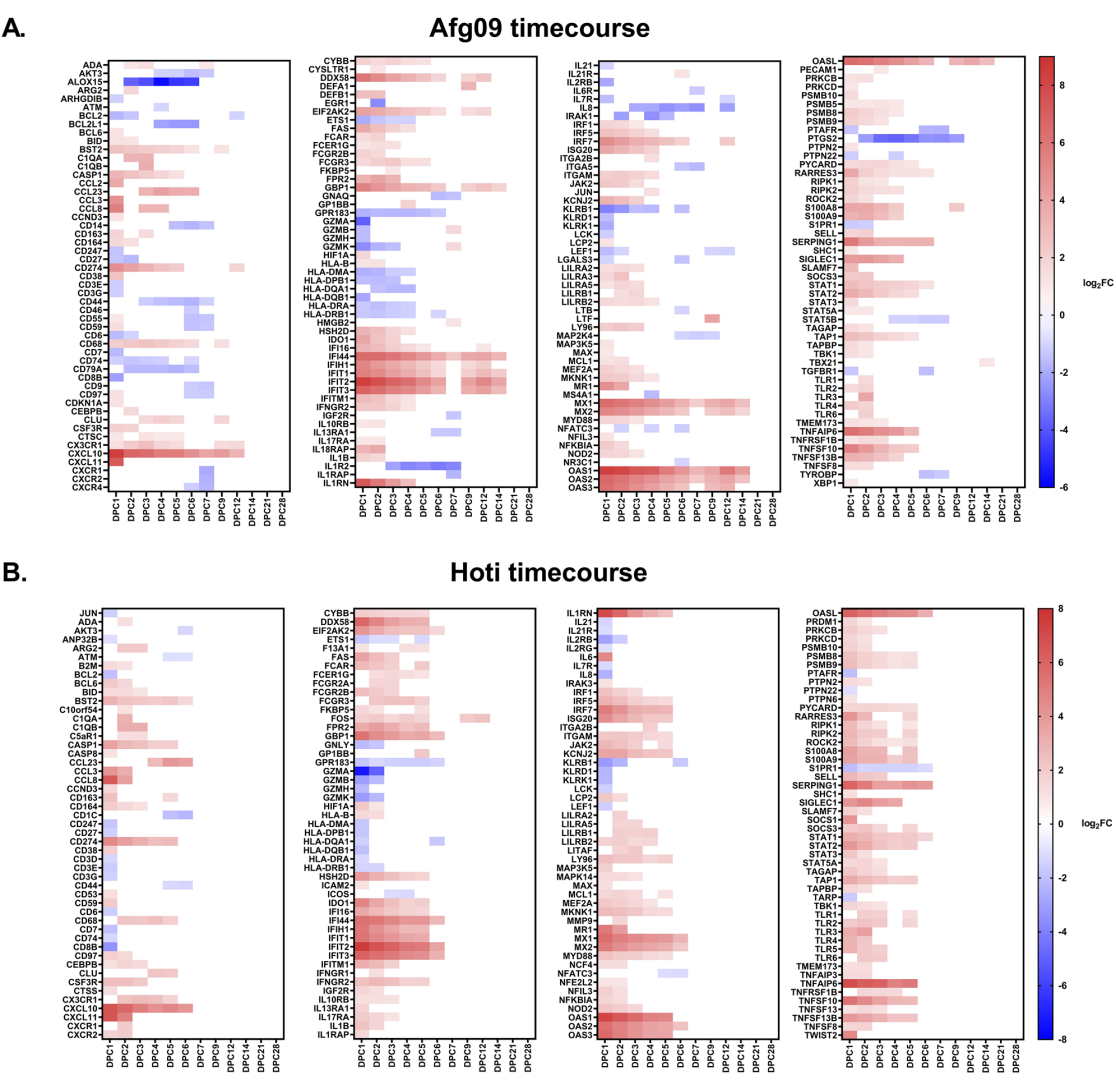
Gene expression changes in NHPs during CCHFV (Afg09 and Hoti) disease. (**A**) Heat map showing expression levels of genes relative to pre-infection levels for Afg09. Only significant genes are shown. (**B**) Heat map showing expression levels of genes relative to pre-infection levels for Hoti. Only significant genes are shown.

The NanoString NHP Immunology codeset contained 754 probes for detecting mRNA of genes related to the immune response of NHPs with previous studies documenting correlation in NHP models of Ebola virus with whole transcriptome sequencing (RNA-seq)^23,24^. Differential gene expression (DGE) analysis using this codeset, performed separately for each viral strain, showed specific expression patterns (Fig. 2A,B). Each timepoint was grouped and compared to day 0 as baseline. Specifically, Afg09 infection induced differential expression of 203 genes while Hoti dysregulated 197 of the 754 genes present in the codeset (Fig. 2A,B). Overall trends of DGE showed upregulation of numerous differentially expressed genes (DEGs) starting at 1 day post-challenge (DPC). Afg09 had a total 150 DEGs (34 downregulated, 116 upregulated) and Hoti had 166 DEGs (40 downregulated, 126 upregulated) at 1 DPC. Hoti infection maintained gene upregulation through 6 DPC, whereas Afg09 did so through 14 DPC. The later DEGs correlate to an adaptive immune response consistent with known disease course of CCHFV infection. For both viruses, early DEGs include many interferon stimulated genes (ISGs) such as *IFIT2*, *OAS1*, *IFIT3*, *IFI44*, *MX1*, and *IFIT1*. CCHFV infection induced strong upregulation for the cytokine genes *CXCL10*, *CXCL11*, *CCL8*, and *IL1RN* with *CXCL10* reaching 8.48 log_2_FC and 6.64 log_2_FC for Afg09 and Hoti, respectively. Analysis of TB-positive and TB-negative NHPs within virus groups and timepoints showed no significant DEGs between the two groups.

### Differences between viral strains

NHP infection with these two viral strains resulted in subtle differences in gene expression. While both strains showed similar numbers of genes differentially expressed at one or more timepoints (203 for Afg09 and 197 for Hoti), Afg09 had DEGs extending for a longer duration than Hoti (Fig. 2A,B). Specifically, 11 Afg09 DEGs remained induced at 14 DPC while Hoti retained induction of only one DEG at 9 DPC and 12 DPC, (*FOS*), with no DEGs after 12 DPC. However, clinical disease courses for the two different viral strains had no statistically significant differences, therefore these transcriptional differences were unexpected.

### Pathway analysis

**Figure 3:**
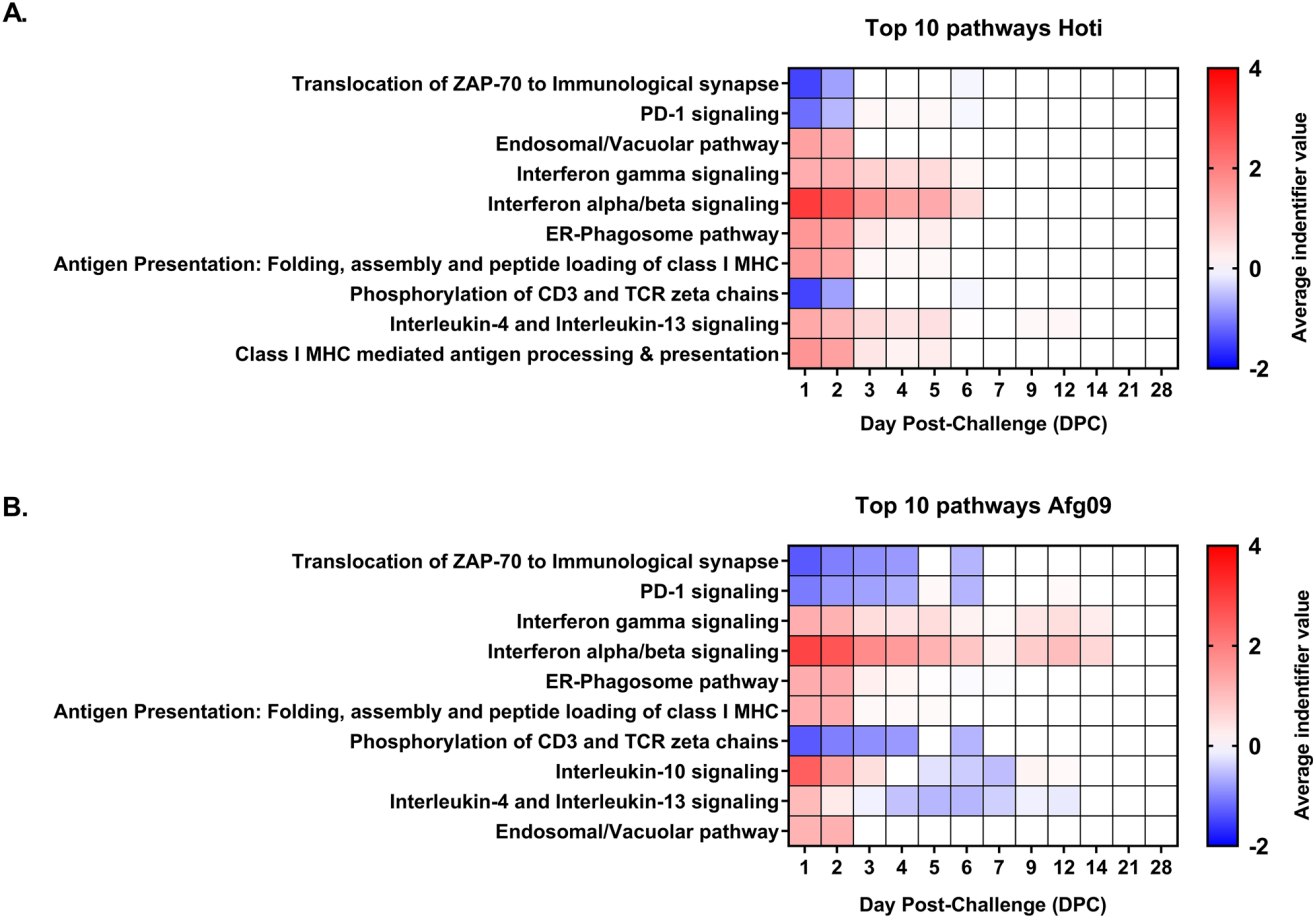
Pathway analysis of DEGs using Reactome. Top 10 pathways for Hoti (**A**) and Afg09 (**B**) are shown here ranked by average identifier value, a consensus of the fold changes of the genes present in the specific pathway. All pathways shown had p-values of 1.11 × 10^–16^.

Analysis with Reactome revealed significantly enriched pathways for both virus groups (Fig. 3A,B). In this analysis, Reactome ranked pathways by *p* value and numerated by the average identifier value, which is a statistical consensus of the fold changes for genes present in those pathways. The top 2 pathways for both virus strains were “Translocation of ZAP-70 to Immunological synapse” and “PD-1 signaling”. In these studies, both had negative average identifier values for the respective strains. Both these pathways play a role in T-cell activation. Lower in the rankings, both viruses downregulated “Phosphorylation of CD2 and TCR zeta chains,” another pathway involved in the activation of T-cells. Both viruses, unsurprisingly, had positive identifier values for Type I and Type II interferon signaling. The “ER-phagosome pathway” and “Antigen Presentation: Folding, assembly, and peptide loading of MHC Class I” pathways being upregulated showed that antigen-presenting cells are potentially actively processing viral antigen for presentation. Differences between viral strains included the presence of “Interleukin-10 signaling” in the Afg09 samples; upregulated early in infection and then downregulated starting at 5 DPC. Both viruses show “Interleukin-4 and interlukin-13 signaling”, however, Hoti showed consistent upregulation while Afg09 changed from upregulation to downregulation at 3 DPC. Additionally, while *IL13* was not a DEG in this dataset, both viruses upregulated *IL13RA1*, a component of the IL-13 receptor. The overall transcriptional response indicated a lack of T-cell interaction early in infection, possibly due to early-onset lymphopenia^22^. There were some cytokine transcripts differentially expressed early in infection (*IL6*, *CXCL10*, and *CCL3* for example), but overall only a minority of DEGs are cytokines (n = 13).

## Discussion

This is the first host transcriptomic study of CCHFV pathogenesis in any primate species. The results presented here showed an overall transcriptional immune response to infection starting 1 DPC and extending to at least 6 DPC for both CCHFV strains investigated. Interestingly, there was a lapse of gene expression changes for the Afg09 group at 7 DPC, which then increased at 9 DPC. As both viremia and clinical score started to wane during 5–7 DPC (Fig. 1B), this may be due to differing recovery times affecting gene expression statistics for the 6 NHPs in that virus strain group. No outliers existed in the 12 NHPs for this timepoint in our analysis. Otherwise, transcriptional responses were broadly similar for both strains, yet there were notable differences in duration of response. Afg09 had a longer duration of DGE, indicating that, at least at the transcriptional level, the NHPs sustained a longer inflammatory and innate immune response for Afg09 compared to Hoti infection. Interestingly, this did not appear to correlate with the observed clinical scores for these two groups as the Hoti challenge group had a longer observed clinical profile versus Afg09. While both challenge groups experienced similar durations of detectable viremia (up to DPC 6), the Afg09 group had more rapid viral kinetics with a peak on DPC 2. It is possible that this faster, more intense viremia may have caused a more prolonged anti-viral gene expression state relative to Hoti, while the slower viremia of the latter sustained a longer clinical window, but triggered a shorter anti-viral transcriptomic response.

The original study showed no measurable impact on clinical outcomes due to latent tuberculosis present in approximately half of the study NHPs. Furthermore, our own gene expression analysis shows no observable difference in the gene activation profiles between TB-positive and negative animals. A similar recently published study comparing Afg09-2990 and Kosova Hoti pathogenesis in cynomolgus macaques^25^ reinforces the relevance of this study’s data, in terms of CCHF clinical pathogenesis and the subsequently derived transcriptomic data. Akin to ours, but notably different from the original, more severe Hoti model published by Haddock et al., these authors also reported a mild disease state for both viral isolates.

Our dataset showed the dysregulation of various cytokines during infection for both viral strains. These analyses implicated many inflammatory cytokines in the pathogenesis of CCFHV similar to documented human cases. To our knowledge, all reported in vivo cytokine data collected from either human or NHP cases of CCHF has been based on detection of soluble protein biomarkers. These cytokine biomarkers include IL-6, IFNA, IFNG, IL-8, TNF, IL-10, IL-12, CXCL1, CXCL10, CCL2, CCL3, CCL4, IL-1RA, IL-1B, IL-4, CSF2, IL-15, and IL-17A^14,16,26^. The present study included gene probes for all of these targets, of which only *IL6*, *IL8*, *CXCL10*, *CCL2*, *CCL3*, and *IL1B* had statistically significant mRNA fold changes. Of those cytokines present in these DEGs, all were upregulated save *IL8*, which was downregulated for both viruses. Of the proposed cytokine biomarkers, *CXCL10* was the most robust DEG in our dataset, reaching 8.48 log_2_FC for Afg09 at 1 DPC and had the longest duration (12 DPC for Afghan and 6 DPC for Hoti). Upregulation of the *CXCL10* encoded protein IP-10 and the *CCL2* encoded protein MCP-1 has been associated with CCHF disease severity and patient outcome in humans, with their serum levels correlating closely with viral load^16,26,27^. Interestingly, transcriptomic evaluation of a transient IFN α/β receptor suppression model of lethal CCHF disease in mice showed similar gene upregulation of some of these same mRNA markers, including *CCL2*, *CCL3*, *CXCL10*, and, *IL1B* during CCHFV infection^28^. Overall, for the 97 probes present in the codeset pertaining to cytokines, combined both viruses had a total of 13 differentially expressed in whole blood over all timepoints. Many of the overall DEGs, such as the *OAS1*, *MX1*, and *IFIT2* are members of the large family of interferon-stimulated genes implicated in the anti-viral defenses of mammalian cells against a wide range of viral pathogens including other hemorrhagic fever viruses like EBOV and LASV^29,30^. Previous studies with human liver cells infected in vitro by CCHFV corroborate many of the ISGs identified in our transcriptomic analysis, further suggesting their relevance for human CCHF disease^31^.

The observed lack of broader inflammatory gene upregulation, beyond activation of the ISG pathway, may be due to the mild disease state of the NHPs in the current study or perhaps be a consequence of sampling bias with our study focused on profiling just whole blood. Indeed, other cell types, such as hepatocytes and endothelial cells, are known cytokine producers in response to viral infection^32,33^. While we noted a brief, upregulation of *IL-6* levels in our samples, this was transient. We also failed to observe any upregulation of *TNFα*. Sustained, high-level expression of both IL-6 and TNFα are associated with CCHF disease, and TNFα in particular is associated with severe, often fatal cases of the disease in humans^26,34^. Interestingly, members of the TNF-superfamily were strongly upregulated in a fatal murine CCHF model^28^. Altogether, our transcriptomic data conforms to the observed clinical data in this NHP study, reinforcing a mild presentation of the disease where a transient viremia activates anti-viral genes involved in the innate immune system. This, however, fails to progress to a more severe phenotype and with it broader activation of a pro-inflammatory state associated with severe disease. Rather, as viral load decreases, innate immune signaling markers like *CXCL10* and ISGs return to basal levels concurrent with a resolution of the clinical phenotype (fever, weight loss, elevated serum biomarkers). DEGs for both viral isolates were consistent with antigen presentation and the emergence of an adaptive immune response to CCHFV challenge; successful anti-CCHFV antibody responses in all challenged animals confirmed these results (Table 1).

In addition to cytokine expression, recent research has indicated additional potential biomarkers for CCHF and other hemorrhagic fever viruses. One such study proposed *HMGB1* as a potential biomarker of severe hemorrhagic fever infection^35^. Here, we found *HMGB2*, a paralog of *HMGB1*, to be upregulated at 7 DPC in Afg09 infection. The lack of *HMGB1* expression may be due to the nature of this animal model not being a severe infection as these animals recovered. Previous studies also implicated VEGF-A, sICAM, TGF-β1, and sVCAM1 as disease biomarkers linked to the severity of CCHFV infection^36–38^. Consistent with that, as all survived infection, NHPs did not show elevated mRNA levels of these biomarkers (*ICAM1*, *VCAM1*) or altered expression of *TGFB1*. While *VEGFA* not included in the codeset, the gene coding the receptor for VEGF-A*, FLT1,* was and did not show elevated gene expression. Similarly, the receptor for TGF-β1, *TGFBR1* appeared downregulated in Afg09 at 1 DPC and 6 DPC. Altogether, this data suggested a lack of activation of pathways associated with severe CCHF pathogenesis.

Pathway analysis indicated, unsurprisingly, an upregulation for both viral strains of “Interferon-gamma” and “Interferon alpha/beta signaling”; important pathways for the host cell antiviral response. The overall transcriptional response indicated a lack of T-cell interaction early in infection, possibly due to early-onset lymphopenia^22^. Further evidencing lack of T-cell involvement, pathways “Translocation of ZAP-70 to Immunological synapse” and “PD-1 signaling” presented as the top 2 pathways for both viral strains with negative identifier values. In addition the pathway “Phosyphorylation of CD3 and TCR zeta chains” had a negative identifier value for both viruses but was ranked 8th for Hoti and 7th for Afg09. These pathways contribute to T-cell activation during infection^39,40^. Overall the pathway analysis was consistent with a mild disease profile distinguished by early T-cell suppression and a moderate inflammatory response culminating in disease resolution.

These data provide a snap shot into the transcriptomic response in whole blood from CCHFV infected NHPs. All NHPs survived, allowing these DEGs to be further investigated as potential prognostic biomarkers. While both the CCHF NHP disease study analyzed here along with a more recently published one were sub-lethal, the earlier reported study by Haddock, et al. displayed a more severe, lethal phenotype^17,25^. In the future, transcriptomic comparisons of mild vs. severe NHP studies could help identify some of the key genes and biological pathways involved in driving severe CCHF pathogenesis, and may aid in the design of useful prognostic assays for triaging mild versus severe cases of CCHF. Furthermore, future collection of transcriptomic data from human CCHF clinical specimens will be key in elucidating the relevant disease biomarkers to observe in NHPs as indicators of severe disease. These could then be used to aid the development of future vaccines and therapeutics for combating this emerging disease.

## Methods and materials

### Ethics statement

As described previously for this animal model experiment^22^, all research was conducted under an IACUC approved protocol in compliance with the following: Animal Welfare Act, PHS Policy, and other Federal statutes and regulations relating to animals and experiments involving animals. The facility where this research was conducted is accredited by the AAALAC, International and adheres to principles stated in the Guide for the Care and Use of Laboratory Animals, National Research Council, 2011, approved USAMRIID animal research protocols undergo an annual review every year. Animals are cared for by a large staff of highly qualified veterinarians, veterinary technicians, and animal caretakers. All personnel caring for and working with animals at USAMRIID have substantial training to ensure only the highest quality animal care and use. NHPs were humanely euthanized by administration of greater than or equal to 6 mg/kg Telazol until a surgical plane of anesthesia was achieved, terminally bled intracardiacly (IC), and administered 0.3–0.4 ml/kg pentobarbital-based euthanasia solution (Fatal-Plus) IC.

### Study design, viral strain, animals, and assays

Twelve cynomolgus macaques were challenged with either Afghan09-2990 or Kosova Hoti strain as previously described and complies with ARRIVE guidelines with additional information included here^22,41^. All animals were serologically naïve for previous CCHFV exposure, and temperatures were monitored continuously by telemetry. Animals were moved in BSL-4 8 days prior to viral challenge for acclimatization and to collect sufficient baseline telemetry data pre-challenge. For the study design, NHPs were randomly divided into 2 groups of 6, each receiving an IV challenge target dose of 6 log_10_ plaque forming units (PFU) virus (actual 6.6 log_10_PFU of Hoti and 6.2 log_10_ PFU of Afg09). Using a one-tailed exact binomial proportion test, the proposed sample size of six animals provides adequate power to estimate the lower bound of the 95% confidence interval of the mortality rate to be 53% when the observed mortality rate is 100% (6/6 dying) compared to the expected population mortality rate constant of 10%. No control group was used as values were compared to animals’ baseline levels. Veterinary personnel were blinded to what animals received which strain of virus. Animals were randomly assigned to cages in the same room. After CCHFV exposure, all animals were monitored for temperature changes, weight loss, survival, and clinical signs. Individual NHP clinical scores were assessed daily and were a composite of animal responsiveness, biscuit/fruit consumption, condition of stool, temperature change from baseline, presence/absence of a rash, bleeding, lymphadenopathy, and dehydration. Blood samples were collected on days 0, 1–7, 9, 12, 14, 21, and 28 for virological, molecular, blood chemistry, immunologic, and hematology analyses. All animals survived the study and were humanely euthanized 35–36 days post exposure.

### Sample collection and preparation

Total RNA extraction methods were described previously in^42^: In summary, all whole blood samples were initially diluted 1:1 with purified molecular grade water prior to being vigorously mixed with a 3:1 ratio of TRIzol LS (ThermoFisher Scientific). All TRIzol LS mixtures and residual matrices were then frozen at − 80 °C until subsequent extraction and/or analysis. RNA for NanoString analysis and qPCR was manually extracted using the miRNeasy kit (Qiagen) following manufacturer’s instructions with the modification that the initial TRIzol-LS/water/whole blood mixture was placed in a Phasemaker phase separation tube (ThermoFisher Scientific) prior to being centrifuged at 12,000×*g* for 15 min at room temperature. The top aqueous layer was then transferred into a miRNeasy kit column.

### NanoString transcriptomic analysis

For host transcriptomic analysis, we employed the nCounter® NHP Immunology Panel (NanoString). Seventy microliters of hybridization buffer was added to the reporter code set to make a master mixture. Eight microliters of this master mixture was then incubated in separate tubes containing 50 ng of extracted host RNA and 2 ul of the capture code set. This hybridization mixture was incubated at 65 °C for 17 h followed by 4 °C until the samples were placed on NanoString Sprint Profiler analysis system. All samples were randomized for processing and technicians were blinded to which samples correspond to which timepoint, NHP, and virus strain. Data was subsequently extracted and analyzed using the Advanced Analysis module on nSolver 4.0.

### NanoString differential gene expression analysis

Gene expression analysis was performed with knowledge of which samples corresponded to which virus strain, NHP, and timepoint. NanoString data processing was described previously in^42^. Resulting gene expression data was processed using the nSolver 4.0 software to perform assay quality assessments, background determination, positive control spike-in normalization, and reference gene normalization. The nCounter Advanced Analysis Module (nCAAM; version 2.0.115) was used to perform initial quality control assessments, reference gene identification, differential gene expression, and other analyses. Quality control (QC) was performed, and samples that did not pass initial QC were re-run on the NanoString nSolver platform. Raw counts were then normalized to geometric mean counts of the synthetic positive controls included on the codeset. nCAAM and the geNorm algorithm was used to normalize each dataset by taking the five most stable housekeeping genes across samples and using it on all datasets. Housekeeping genes used are listed in Supplementary File 1. The “Optimal” setting was used with nCAAM to calculate differential gene expression using the day 0 baseline as the reference group for each time point. Cutoff for differential gene expression was ± 1 log_2_fold-change with a Benjamini–Yekutieli-adjusted *p* value of less than or equal to 0.05. The background count threshold was 2 × the standard deviation (SD) of the mean count of all synthetic negative control probes present on the codeset. Normalized count data and DEGs can be found in Supplementary Files 2, 3, and 4. Data files are found under GEO accession GSE169698.

### Pathway analysis

The Reactome database was used to determine significantly enriched pathways for both viruses (www.reactome.org)^43^. Reactome data and heat maps were graphed using GraphPad Prism 8.3.1.

## Supplementary Information

Supplementary Information 1.

Supplementary Information 2.

Supplementary Information 3.

Supplementary Information 4.

## Data Availability

All data generated or analyzed during this study are included in this published article (and its Supplementary Information files) and found under GEO accession GSE169698.
